# Measuring health‑ and oral health-related quality of life in secondary school pupils: a head‑to‑head psychometric comparison of CHU9D and CARIES-QC-U

**DOI:** 10.1186/s12903-025-07467-0

**Published:** 2025-12-23

**Authors:** Helen J. Rogers, Anju Keetharuth, Nicola Innes, Zoe Marshman

**Affiliations:** 1 School of Dental Sciences, Faculty of Medical Sciences, Newcastle University, Newcastle-upon-Tyne, United Kingdom; 2 School of Medicine and Population Health, University of Sheffield, Sheffield, United Kingdom; 3 School of Dentistry, College of Biomedical and Life Sciences, Cardiff University, Dental Drive, Heath Park , Cardiff, United Kingdom; 4 School of Clinical Dentistry, University of Sheffield, Sheffield, United Kingdom

**Keywords:** Caries, QALYs, Utilities, Child oral health

## Abstract

**Objectives:**

Dental caries impacts children’s health- and oral health-related quality of life. Preference-based measures (PBM) can quantify these impacts as utilities, facilitating economic evaluation of interventions. Two paediatric PBMs (one generic (CHU9D) and one condition-specific (CARIES-QC-U)) were used in the BRIGHT randomised control trial investigating the impact of a behaviour change intervention on schoolchildren’s oral health. No comparison has been made of these two instruments previously. This study aimed to compare the psychometric properties of CHU9D and CARIES-QC-U using trial data.

**Methods:**

Baseline trial data were assessed. Mean utility scores, missing values and floor and ceiling effects were determined for each instrument. Cronbach’s alpha was assessed to indicate internal consistency for each instrument. Correlations were explored between CARIES-QC-U and CHU9D, the dimensions within the two instruments, and between each instrument and DMFT. Effect sizes (Cohen’s *d*) were explored for each component of DMFT in relation to overall utility values from each instrument.

**Results:**

Baseline data from 4542 schoolchildren aged 11–13 years were analysed. Over a third of participants had obvious caries experience. Mean utility scores for CARIES-QC-U and CHU9D were 0.76 and 0.91 respectively. Missing data was low for both instruments. Floor and ceiling effects were greater for CARIES-QC-U. Internal consistency was acceptable for both instruments. Correlation between utilities of CARIES-QC-U and CHU9D was weak at 0.35. Correlation between clinical caries experience and utilities from CARIES-QC-U was negative (*r*=-0.09) and stronger than with CHU9D (*r*=-0.02). Correlations between dimensions within the instruments were weaker than anticipated. Small, statistically significant effects were seen for both instruments and the decayed (D) component of DMFT, though this was stronger with CARIES-QC-U.

**Conclusions:**

The burden of caries was reflected in participant utility scores. Whilst both PBMs performed well psychometrically, CARIES-QC-U demonstrated greater ability to capture impacts related to dental caries, indicating better suitability for caries research than CHU9D.

**Supplementary Information:**

The online version contains supplementary material available at 10.1186/s12903-025-07467-0.

## Background

The poor oral health of children in the UK has attracted significant public, media and political attention. The most recent epidemiological study in England, Wales and Northern Ireland found approximately one third of 12-year-olds (34%) had obvious decay experience, with this increasing to almost a half of 15-year-olds (46%)[1]. There is a wealth of evidence to demonstrate the negative effect of caries on children’s lives, causing toothache, difficulty sleeping and eating, with further impacts on their overall growth and development [2–4]. 

The term quality of life is a broad, multi-dimensional concept, encompassing health and social wellbeing, alongside more diverse non-health domains such as the economic and political aspects of a person’s life [5]. Most clinical researchers are concerned with those aspects of quality of life related to health, thus the notion of health-related quality of life is a primary focus [6]. A number of instruments have been designed to measure health-related quality of life (HRQoL) in children, and one in particular, the Child Health Utility 9 Dimensions (CHU9D) has actively involved children during development [7–9]. Many of these instruments, including CHU9D, are known as utility measures or preference-based measures, as they have a scoring system (or tariff) that is derived from a preference-elicitation survey, typically undertaken with a sample of the general population using health state valuation methods such as standard gamble, time trade off or more novel techniques such as discrete choice experiments. This enables them to generate Quality Adjusted Life Years (QALYs), which combine the quality and length of life gained as a result of a healthcare intervention into a single standardised unit, for use in economic evaluations of such interventions. These differ from non-preference-based measures, which typically have simple summative scoring systems, and hence have a more limited use in economic evaluation.

Since the impact of dental diseases such as caries extend beyond the mouth, oral health-related quality of life (OHRQoL) has been defined as “*the impact of oral disease and disorders on aspects of everyday life that a patient or person values*,* that are of sufficient magnitude*,* in terms of frequency*,* severity or duration to affect their experience and perception of their life overall.”*[10] There are a range of non-preference-based measures available that have been developed to capture children’s OHRQoL, and the impact of interventions to treat them [11]. Nonetheless, the majority of these are generic in nature, many have been found to have inherent limitations, and the ability of these instruments to capture the full range of impacts of childhood caries has been queried [11, 12]. In response to this, a caries-specific measure of OHRQoL known as CARIES-QC, was developed and evaluated with involvement of children at every stage [13]. This was subsequently adapted to form a utility version (CARIES-QC-U) that is capable of generating caries-specific QALYs for use in economic evaluation of interventions to improve children’s oral health [14–16]. This adaptation was undertaken to address the findings of a systematic review, which identified a lack of high quality economic evaluations in child oral health research, potentially attributable to a deficiency of appropriate utility instruments with which to conduct cost-utility analysis [17]. 

To date, the CHU9D has been used in a number of oral health studies[18, 19], including a split-mouth randomised controlled trial in New Zealand which sought to evaluate its responsiveness to changes in the oral health of children receiving dental care over a 12 month period [20]. In comparison, whilst the original CARIES-QC instrument has been used widely[21–28], the CARIES-QC-U utility tariffs have only recently begun to be applied to these results [29]. To our knowledge, to date, there has been no direct comparison of the psychometric properties of the two utility instruments using data from a UK adolescent population.

The recently completed BRIGHT trial assessed whether a school-based lesson together with text messages to support toothbrushing could prevent dental caries in adolescents from deprived areas. It was novel as it employed both the CHU9D and CARIES-QC, with subsequent application of the utility tariffs from CARIES-QC-U[29, 30]. This paper aims to investigate the psychometric performance and differences in utilities generated by the CHU9D and CARIES-QC-U instruments using the baseline data obtained from secondary school children participating in the BRIGHT trial.

## Methods

This study centred on secondary analysis of the baseline dataset from the BRIGHT trial.

### Dataset

The BRIGHT trial (ISRCTN12139369) was a multi-centre, assessor blinded, two-arm cluster randomised control trial conducted across England, Scotland and Wales. State-funded secondary schools with above average proportions of pupils eligible for free school meals (an indicator of deprivation, which has a strong association with caries) took part. Pupils in two separate year groups aged 11–12 (Year 7/Senior 1) and 12–13 years (Year 8/Senior 2) were recruited, and each year groups randomised 1:1, to either the intervention (a school-based lesson and twice daily SMS reminders to brush their teeth) or the control (routine education and no SMS).

The primary outcome was determined at the child level, as the presence of at least one treated or untreated carious lesion extending into dentine at 2.5 years, recorded using the Decayed Missing and Filled Teeth (D_4 − 6_MFT) index. Secondary outcomes included HRQoL (measured using CHU9D) and OHRQoL (measured using CARIES-QC).

The dataset used for this paper includes baseline data from 4542 participants for whom utilities could be calculated for both measures. Participant characteristics are shown in Table 1.

**Table 1:** Baseline characteristics of participants in the BRIGHT trial

Characteristic	**Baseline characteristics**	
	**Intervention arm (n= 2201)**	**Control arm (n= 2341)**
Age, mean (SD), years	12.8 (0.7)	12.6 (0.6)
School year, *n* (%)		
Year 7/Senior 1	1009 (45.8)	1539 (65.7)
Year 8/Senior 2	1192 (54.2)	802 (34.3)
Sex: female, *n* (%)	1185 (53.8)	1283 (54.8)
Eligible for free school meals, *n* (%)	494(23.2)	495 (22.1)
Presence of D_4-6_MFT *n *(%)	758 (34.5)	816 (34.9)
Number of D_4-6_MFT per pupil		
Mean (SD)	0.76 (1.41)	0.77 (1.35)
Mean (SD)Median (IQR)	0.0 (0.0, 1.0)	0.0 (0.0, 1.0)
Number (mean [SD]) of:		
D: decayed teeth (into dentine)	0.25 (0.75)	0.29 (0.78)
M: teeth extracted due to caries	0.11 (0.60)	0.07 (0.44)
F: filled teeth	0.41 (0.92)	0.41 (0.92)0.41 (0.91)

D_4-6_MFT refers to the combined number of permanent teeth with evident decay into dentine, missing permanent teeth and filled (restored) permanent teeth

### Instruments

#### CHU9D

The CHU9D was designed for 7–17 year-olds, and consists of nine dimensions: ‘*worried’*,* ‘sad’*,* ‘pain’*,* ‘tired’*,* ‘annoyed’*,* ‘schoolwork/homework’*,* ‘sleep’*,* ‘daily routine’*, and *‘activities’*. Each dimension is represented by a single question with five ordinal response options (*‘I don’t feel worried today’; I’ feel a little bit worried today’; ‘I feel a bit worried today’; ‘I feel quite worried today’; ‘I feel very worried today’*)[7, 31]. The recall period is today/last night. The responses can be taken together as a description of the HRQoL of the child, known as a health state. The CHU9D descriptive system (see supplemental material) comprises a broad range of health states (due to the different combinations of response options on each of the nine dimensions), and each unique health state has a preference weight associated with it. These preference weights give a utility value (on a 0–1 scale, where 1 is perfect health and 0 is a state equivalent to being dead) which, when combined with the length of life, enables the generation of QALYs. These preference weights were derived from UK adults using standard gamble and ranking valuation methods [9]. 

#### CARIES-QC-U

The CARIES-QC-U was developed and evaluated for 5–16 year-olds and comprises five items: *‘hurt’*,* ‘annoy’*,* ‘kept awake’*,* ‘difficulty eating’* and *‘cry’*(see supplemental material) [14]. Each item has three different response levels (*‘a lot’; ‘a bit’; ‘not at all’*). CARIES-QC-U has not been evaluated for use as a standalone five-item questionnaire, and hence the original 13-item CARIES-QC instrument should be completed by participants, whereby their responses to these five questions only will describe their oral health state. No recall period is defined for completion of CARIES-QC[13]. CARIES-QC-U has two value sets which give utility values derived from adults and adolescents respectively [32]. The adolescent value set was used for this study, which gained preferences from 10–16 year-olds using best-worst scaling in a UK-wide online survey [32]. 

### Analysis

The following classical psychometric tests were undertaken on the BRIGHT trial dataset to compare the properties of the CHU9D and CARIES-QC-U instruments.

### Acceptability

The mean utility values, distribution of responses, the proportion of missing values and floor (percentage with lowest possible score) and ceiling effects (percentage with highest possible score) were reported to assess the acceptability of the two instruments. Missing values over 5% were considered to indicate reduce acceptability of an instrument to the adolescents participating in the BRIGHT trial and could be indicative of difficulties in comprehension [33]. Floor and ceiling effects were deemed to be present if more than 15% of participants reported the lowest possible score, or the highest possible score [34]. An instrument with strong floor or ceiling effects could be considered to have a reduced sensitivity to changes in oral health status. Nonetheless, it is acknowledged that floor and ceiling effects would be more likely to be present in CARIES-QC-U, which has only three response options, compared to the CHU9D which has five.

## Internal consistency

Cronbach’s alpha (α) to assess the internal consistency reliability for each instrument, where α >0.7 and >0.8 was identified as acceptable and good respectively [35]. A higher level of internal consistency suggests that the instrument contains a closely related set of items, which are measuring the same concept.

### Construct validity

#### Convergent validity

Convergent validity between utility scores and items was evaluated by investigating a priori hypothesised relationships using Pearson and Spearman’s correlation coefficients respectively where *r* ≥ 0.7, strong; *r* >0.5, moderate; *r* >0.2, weak [35]. At the level of utility scores, a strong correlation was hypothesised between DMFT_4 − 6_ and CARIES-QC-U and a moderate correlation between DMFT_4 − 6_ and CHU9D. These correlations are expected to be negative, as utility values are anticipated to decrease in reflection of deteriorating oral health as the dental disease observed with the DMFT_4 − 6_ index increases. A moderate correlation between utility scores from CARIES-QC-U and CHU9D was also anticipated.

At item levels, moderate correlations were hypothesised between the following dimensions of CARIES-QC-U and CHU9D respectively: *‘cried’* and *‘sad’; ‘hurt’* and *‘pain’; ‘awake’* and *‘tired’; ‘annoy’; ‘awake’* and *‘sleep’; ‘hard to eat’* and *‘daily routine’.*

A Bland Altman plot was generated to assess agreement between the two instruments, whereby the closer the mean difference is to zero, the greater the agreement [36, 37]. 

#### Known group differences

Known group differences in relation to the overall utility values gained with each instrument were explored for the following “groups”: those with decayed (D), missing (M) and filled (F) components of the DMFT_4 − 6_ and those eligible for free school meals. It was anticipated that the filled (F) component of the DMFT_4 − 6_ index would have a smaller effect size than the decayed (D) and missing (M) components. Participants eligible for free school meals were hypothesised to have lower utility values. It was hypothesised that those who reported higher level of problems with their teeth would also have lower utilities on both instruments.

Cohen’s *d* is a frequently used effect size index, which is found by subtracting one mean from another and dividing the result by the pooled standard deviation of the groups. Eta-squared was calculated in the instance where there were more than two groups. These effect sizes were interpreted using Cohen’s criteria, whereby < 0.2 is deemed inconsequential, 0.2–0.5 is considered small, 0.5–0.8 is considered moderate and above 0.8 is considered large [38]. Statistical significance was assessed at the 5% level.

## Results

The baseline characteristics of participants in the BRIGHT trial are shown in Table 1.

### Acceptability

Mean utility scores for CARIES-QC-U and CHU9D were 0.76 and 0.91 respectively. Table 2 shows a broader distribution of responses seen for CARIES-QC-U, with minimum utility values below zero, suggesting a health state considered to be worse than death. Missing data was low for both instruments at 1.79% for CARIES-QC-U, and 1.97% for CHU9D. Floor and ceiling effects were considerably greater for CARIES-QC-U, as anticipated due to fewer response levels.

### Internal consistency

The Cronbach alpha (α) for CARIES-QC-U and CHU9D was acceptable at 0.73 and 0.75 respectively (Table 2).

**Table 2:** Distribution of participant responses and internal consistency reliability of the CARIES-QC-U and CHU9D responses

	Mean	SD	Median	Min	Max	Floor effect %	Ceiling effect %	Cronbach alpha (α)
CARIES-QC-U	0.76	0.28	0.86	−0.33	1	0.07	43.93	0.73
CHU9D	0.91	0.09	0.93	0.35	1	0.02	18.49	0.75

### Construct validity

#### Convergent validity

The correlation between the utilities of CARIES-QC-U and CHU9D was weak but statistically significant at 0.35. The correlation between the clinical variable DMFT_4 − 6_ and utility values derived from CARIES-QC-U was statistically significant negative and stronger (*r* = −0.09) than the correlation between DMFT_4 − 6_ and CHU9D (*r* = −0.02) and not statistically significant, as anticipated. The Spearman correlations between the items of the two measures were weak and ranged from 0.06 to 0.34 (Table 3). The highest correlations were observed between the pain dimension of CHU9D and the five items within CARIES-QC-U. All correlations were weaker than hypothesised.

**Table 3:** Spearman correlations between the items within CARIES-QC-U and CHU9D

CHU9D items	CARIES-QC-U items				
	Hurt	Annoy	Awake	Hard to eat	Cried
Worried	0.17	0.25	0.14	0.16	0.22
Sad	0.16	0.18	0.14	0.13	0.19
Pain	0.34	0.32	0.28	0.28	0.24
Tired	0.14	0.16	0.06	0.13	0.09
Annoyed	0.18	0.21	0.14	0.15	0.15
School work	0.16	0.20	0.15	0.17	0.13
Sleep	0.16	0.19	0.17	0.18	0.14
Daily routine	0.18	0.21	0.19	0.22	0.16
Activities	0.13	0.16	0.12	0.14	0.13

**Fig. 1:**
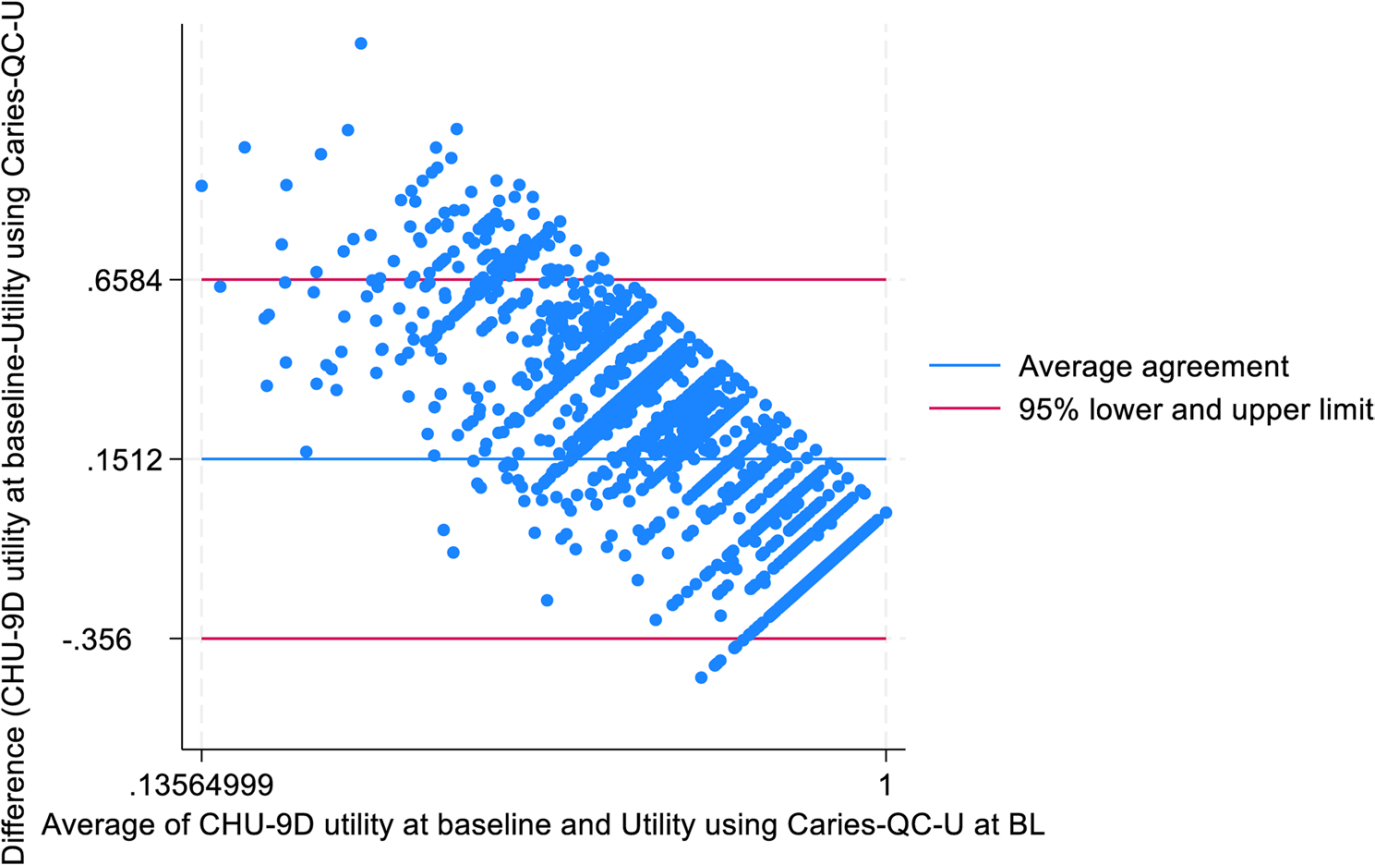
Bland Altman plot demonstrating the differences in utilities derived from CARIES-QC-U and CHU9D against the average of utilities from the two instruments at baseline

A Bland Altman plot (Fig. 1) indicates a mean (SD) difference of 0.15 (0.26) between CARIES-QC-U and CHU9D, with wide limits of agreement. The pattern in the scatter of points indicates consistent bias.

#### Known group differences

A small, but statistically significant effect size was seen for CARIES-QC-U and the decayed (D) component of DMFT_4 − 6_. As anticipated, this effect size reduced further for the missing (M) and filled (F) components respectively in relation to CARIES-QC-U. The effect size for CHU9D and the decayed component of DMFT_4 − 6_ was smaller than seen with CARIES-QC-U, though was still statistically significant. Interestingly, a greater effect size was noted for the missing (M) component and CHU9D, compared to the decayed (D) component, which was not expected. Also, a negative effect size was seen for CHU9D and the filled (F) component. The further effect sizes reveal that CARIES-QC-U is better at detecting the differences among the other known groups. These are shown in Table 4.

**Table 4:** Effect sizes for D_4–6_MFT components in relation to CARIES-QC-U and CHU9D

Groups with a priori known differences		Effect size		*p* value	
D_4 − 6_ MFT component					
	*n*	CARIES-QC-U	CHU9D	CARIES-QC-U	CHU9D
D = 0	3812	0.208	0.095	< 0.001	0.031
D > 0	730				
M = 0	4346	0.161	0.129	0.035	0.103
M > 0	196				
F = 0	3493	0.116	−0.046	0.002	0.187
F > 0	1049				
Eligible for free school meal					
No	3384	0.160	0.015	< 0.001	0.686
Yes	989				
Global question from CARIES-QC (How much was of a problem are your teeth for you?					
Not at all	2487	0.221	0.062	< 0.001	< 0.001
A bit	1878				
A lot	164				

Some participant totals add up to < 4542 because of missing data for the known group

## Discussion

This is, to our knowledge, the first study to compare the psychometric performance of the CARIES-QC-U and CHU9D instruments. The findings suggest that both instruments appear to perform well psychometrically in terms of acceptability and internal consistency, though are clearly measuring different constructs. The burden of caries was high in the study sample, which was reflected in the utility values from both instruments, particularly CARIES-QC-U. Whilst the correlation between CARIES-QC-U and clinical indicators of oral health (D_4 − 6_MFT) was weak, it was stronger than that seen with CHU9D. Effect sizes highlight discrepancies in the alignment of CHU9D with individual components of the (D_4 − 6_MFT) index, and confirm the superiority of CARIES-QC-U in this regard. The results indicate that CARIES-QC-U is more suited to assessment of OHRQoL and should be used to generate utilities in favour of CHU9D in economic evaluations of interventions to improve children’s oral health.

The CHU9D instrument has been employed in a number of studies investigating children’s oral health. The active involvement of children in the development of CHU9D is one of its advantages over other generic paediatric preference-based measures[7, 31], such as EQ-5D-Y[39], HUI2[40] and HUI3 [41]. This contributed to its selection for use in this oral health research of children and young people, as well as the fact that, prior to the development of CARIES-QC-U, there was no condition-specific instrument available [17]. Nonetheless, a number of issues have been highlighted regarding the use of CHU9D in this field. In the aforementioned trial based in New Zealand, the CHU9D was found to be unresponsive to changing components in children’s dental caries status [20]. Furthermore, a comparison of the Dutch versions of CARIES-QC and CHU9D in a study of 11-year old children across 4 cities in the Netherlands revealed that the CHU9D was not found to correlate with any of the clinical data [42]. 

In this study using BRIGHT trial data, a negligible correlation was found between CHU9D and the overall DMFT, which was weaker than that of CARIES-QC-U. Interestingly, a statistically significant effect size was seen between CHU9D and the decayed component of D_4 − 6_MFT, though again this was not as significant or as strong as that of CARIES-QC-U and the decayed component. Furthermore, the CHU9D instrument did not function as anticipated with respect to the other components of the D_4 − 6_MFT index. For example, it was found to have a negative effect size with filled teeth, and the effect size for missing teeth was higher than what was found for decayed teeth, which would contradict our expectations.

All correlations between the items in CARIES-QC-U and CHU9D were weaker than anticipated. This was particularly interesting for the items from each instrument related to feeling ‘annoyed’, which would be expected to have at least a moderate correlation as they are almost identical. Potentially some slight differences in the wording in the questions, or the context amongst the other questions within the respective questionnaires may have affected children’s interpretation. Similarly, whilst the correlations between CARIES-QC-U and D_4 − 6_MFT were in the expected direction and statistically significant, the magnitude of this relationship was weaker than anticipated. This could potentially be explained by the high prevalence and severity of caries in this age group, as extraction of first permanent molars may have already been undertaken, and caries had yet to develop in the premolars. Importantly, the BRIGHT trial did not document caries prevalence in primary teeth, which most of the study population would still have retained at baseline [29]. It is possible that children’s responses to the CARIES-QC questionnaire (hence contributing to the CARIES-QC-U utility score) could have encompassed impacts from primary teeth that were not documented in the clinical examinations. Similarly, whilst CARIES-QC and CARIES-QC-U are intended to be caries-specific, they may still capture impacts from other oral health conditions. In this age group, it is possible that concerns about malocclusion may have contributed to children’s responses.

This study has a number of limitations. Firstly, the recall period for the two instruments differed, which may have influenced participant responses and could explain some of the variation in findings from the two instruments. Furthermore, the comparison of floor and ceiling effects between the instruments was notably affected by differences in the number of response levels. Finally, the aforementioned omission of caries documentation in the primary dentition may have led to an underestimation of the magnitude of relationship between clinical findings and the utility scores.

Interestingly, since the introduction of CARIES-QC-U, two further oral health-specific utility instruments have been developed [43, 44]. One of these, the ECOHIS-4D, derived from the original 13-item ECOHIS OHRQoL instrument and validated for proxy completion on behalf of children aged three to five years[43], has been compared to the CHU9D instrument [45]. The authors found the ECOHIS-4D to be more sensitive in assessing the OHRQoL of children with early childhood caries, compared to CHU9D, reinforcing the findings from the present study that CHU9D is not the best choice of utility instrument for oral health research [45]. 

With greater availability of psychometrically sound, validated oral health- and caries-specific preference-based measures for paediatric populations than ever before, it is important that further research focuses on implementation of these instruments to address the ongoing lack of economic evaluations of interventions to improve children’s oral health.

## Supplementary Information

Supplementary Material 1.

## Data Availability

The full report for the BRIGHT trial can be found at the link on the NIHR HTA website. [https://www.fundingawards.nihr.ac.uk/award/15/166/08](https:/www.fundingawards.nihr.ac.uk/award/15/166/08). The dataset used in this study is available from the authors of the BRIGHT trial upon reasonable request.
