# Supplementary material for: Measuring health‑ and oral health-related quality of life in secondary school pupils: a head‑to‑head psychometric comparison of CHU9D and CARIES-QC-U
Source: BMC Oral Health. 2025 Dec 23;26:167. doi: 10.1186/s12903-025-07467-0 (PMC12837086; doi:10.1186/s12903-025-07467-0)
Supplement: Supplementary file 1 — Supplementary Material 1. [file 12903_2025_7467_MOESM1_ESM.docx]

**Supplemental Material**

**Descriptive system for CHU-9D instrument, from Rowen et al, 2018**


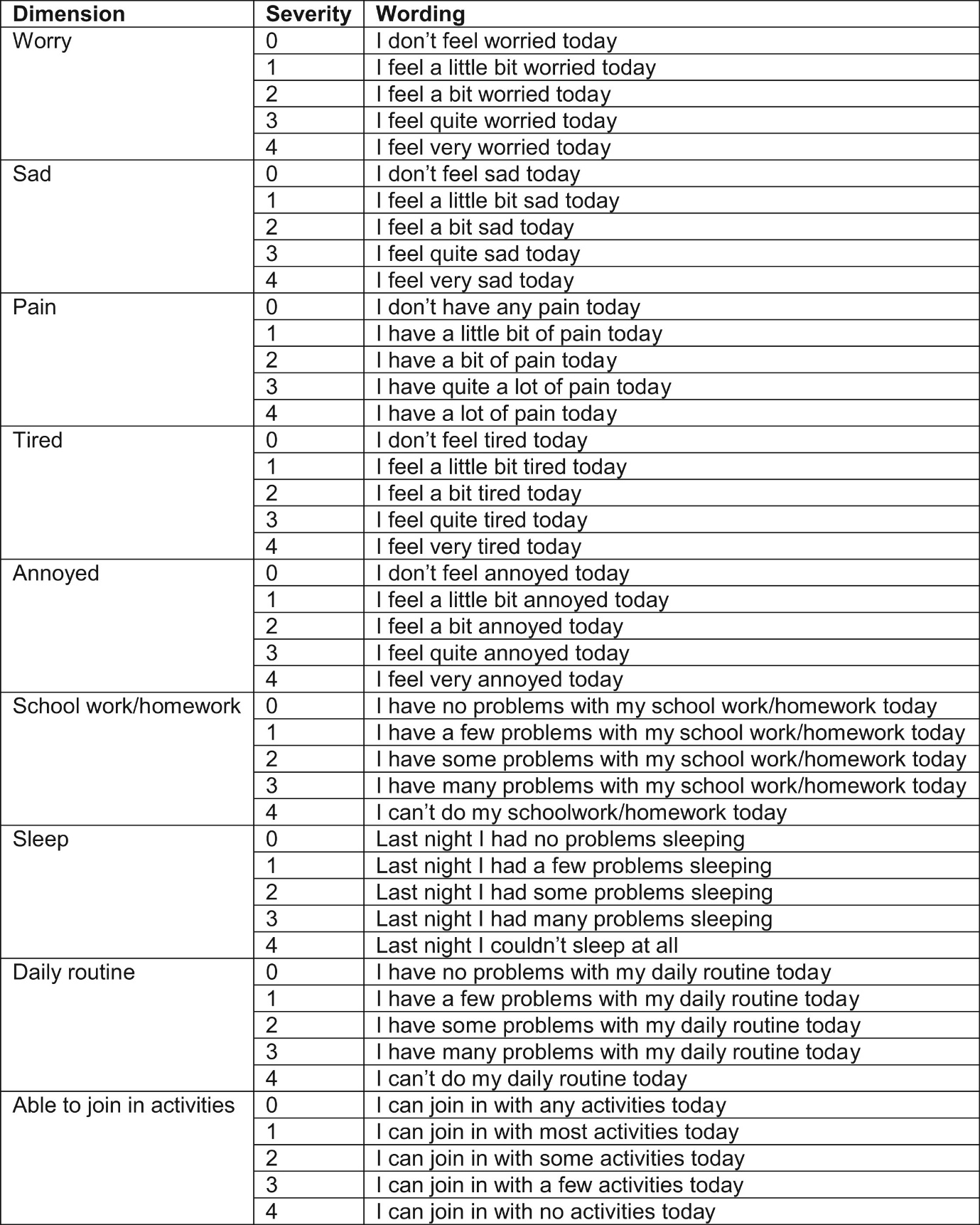


**Descriptive system for CARIES-QC-U instrument, from Rogers et al, 2020**

| **Items** | **Severity** | **Health state descriptors** |
| --- | --- | --- |
| **Hurt** | 0  1  2 | My teeth do not hurt me at all  My teeth hurt me a bit  My teeth hurt me a lot |
| **Annoy** | 0  1  2 | My teeth do not annoy me at all  My teeth annoy me a bit  My teeth annoy me a lot |
| **Kept awake** |  | My teeth do not keep me awake at all  My teeth keep me awake a bit  My teeth keep me awake a lot |
| **Hard to eat** |  | My teeth do not make it hard to eat some foods  My teeth make it a bit hard to eat some foods  My teeth make it really hard to eat some foods |
| **Cried** |  | My teeth do not make me cry at all  My teeth make me cry a bit  My teeth make me cry a lot |
